# Complete Genome Sequence of Human Adenovirus Type 55 Associated with Acute Respiratory Disease, Isolated from a Military Base in the Republic of Korea

**DOI:** 10.1128/genomeA.01565-16

**Published:** 2017-03-09

**Authors:** Se Hun Gu, Dong Hyun Song, Daesang Lee, Kyungmin Huh, Hongseok Yoo, Hong Sang Oh, Jaehun Jung, Koung In Woo, Mirang Kim, Woong Seog, Il-Ung Hwang, Seong Tae Jeong

**Affiliations:** a 5th R&D Institute, Agency for Defense Development, Yuseong, Daejeon, Republic of Korea; b Armed Forces Capital Hospital, Bundang-gu, Republic of Korea; c Armed Forces Medical Command, Bundang-gu, Republic of Korea

## Abstract

Human adenovirus (HAdV) (genus *Mastadenovirus*; family *Adenoviridae*) serotype 55 is a reemerging pathogen associated with acute respiratory disease. Here, we report the complete genome sequence of HAdV-55 strain AFMC 16-0011, isolated from a military recruit, using next-generation sequencing technology.

## GENOME ANNOUNCEMENT

Adenoviruses are double-stranded DNA viruses that belong to the *Adenoviridae* family, which comprises five genera. Human adenoviruses (HAdVs) are classified in the *Mastadenovirus* genus. There are more than 68 HAdV genotypes, characterized by neutralization assay, which are grouped within seven species (A to G) (1–5). HAdV species B, C, and E (4) are associated with acute respiratory disease (ARD). HAdV serotype 55 has been responsible for ARD outbreaks in military camps in Spain, Turkey, China, Singapore, and Israel (2, 6–9). Recently, HAdV-55 was identified as the cause of an outbreak of febrile respiratory infections in the Republic of Korea (Yoo et al., unpublished data). Here, we report the full-length genome sequence of HAdV-55 strain AFMC 16-0011, isolated from the throat swab of a military recruit in the Republic of Korea.

The whole-genome sequence of HAdV-55 strain AFMC 16-0011 was sequenced using next-generation sequencing (NGS) technology. DNA was sheared using an M220 focused-ultrasonicator (Covaris, Woburn, MA, USA). The library was prepared using the TruSeq Nano DNA sample preparation kit (Illumina, San Diego, CA, USA). Deep sequencing was performed on a MiSeq benchtop sequencer (Illumina) using a MiSeq reagent kit version 2 (Illumina) with 2 × 150-bp paired-end reads. All reads with quality scores >Q20 score were trimmed for reference mapping and consensus sequence extraction using CLC Genomics Workbench version 7.5.2 (CLC Bio, Aarhus, Denmark). NGS data of the HAdV-55 strain AFMC 16-0011 generated 19,831 reads with an average read length of 150 bp. Gaps at the 5′ and 3′ termini were filled by designing specific primers, using the Sanger sequencing method. The full-length genome of HAdV-55 strain AFMC 16-0011 was 34,776 nucleotides with 48.77% G+C content. The inverted termini repeat sequences were 137 bp in length. A comparison of the whole-genome sequences of HAdV-55 strain AFMC 16-0011 and HAdV-55-B/CHN/BJ01/2011/55 (P14H11F14) (GenBank accession no. JX491639) from China showed 99.93% sequence similarity at the nucleotide level. The whole-genome sequencing of HAdVs will accelerate the acquisition of new knowledge about their phylogenetic, phylogeographic, and evolutionary origin.

### Accession number(s).

The whole-genome sequence of HAdV-55 strain AFMC 16-0011 was deposited in GenBank under the accession number KX494979.
